# Incidental Finding of an Intratendinous Supraspinatus Cyst

**DOI:** 10.7759/cureus.72541

**Published:** 2024-10-28

**Authors:** Wan Lye Cheong, Mohd Nizlan Mohd Nasir, Raymond D. K. Yeak, Johan Abdul Kahar

**Affiliations:** 1 Orthopaedics, Universiti Putra Malaysia, Serdang, MYS; 2 Orthopaedics, Sports Surgery Division, Universiti Putra Malaysia, Serdang, MYS

**Keywords:** arthroscopy, intratendinous cyst, right shoulder pain, rotator cuff, shoulder, supraspinatus tendon

## Abstract

We present the case of a 32-year-old male patient with an intratendinous cyst of the supraspinatus tendon identified during shoulder arthroscopy. The patient presented with right shoulder pain, worsened by shoulder flexion and abduction, after playing darts. There was no history of trauma. Initial clinical examination revealed only slightly reduced subscapularis muscle strength and tenderness over the long head of the biceps tendon. Magnetic resonance imaging showed subacromial impingement, a bursal side partial tear of the supraspinatus tendon, and subscapularis tendinosis. Despite physiotherapy, which resulted in a full shoulder range of motion and negative provocative tests, his symptoms persisted for two years. He subsequently underwent a diagnostic arthroscopy, subacromial decompression, and biceps tenodesis. Intra-operatively, an intratendinous cyst within the supraspinatus tendon was identified and debrided, explaining the persistence of his symptoms. Six months postoperatively, he maintained a full shoulder range of motion with complete resolution of symptoms.

## Introduction

Shoulder periarticular cysts are occasionally identified during investigation for shoulder pain [1]. They are often discovered incidentally on routine magnetic resonance imaging (MRI) conducted for rotator cuff tears or shoulder impingement [2]. Rarely, these cysts are identified only during shoulder arthroscopy performed for diagnostic purposes or rotator cuff repair.

Depending on their size and location, they may be asymptomatic or produce pain and mechanical nerve entrapment [3]. The most common types of periarticular shoulder cysts are paralabral cysts and intramuscular rotator cuff cysts. These cysts often arise after acute trauma or repetitive motion injuries. Paralabral cysts are juxta-articular lesions frequently associated with labral tears [1]. An enlarging paralabral cyst located in the suprascapular or spinoglenoid notch may compress the suprascapular nerve, leading to pain and weakness in shoulder abduction and external rotation [4]. Additionally, fluid collections around the shoulder can be linked to degenerative joint conditions, such as those seen in acromioclavicular osteoarthritis, presenting as a supraclavicular mass. Rotator cuff injuries may also lead to the formation of intramuscular cysts, which can be subtle on MRI and may be missed during diagnostic arthroscopy [5].

These cysts, especially when small or atypical, may be overlooked, and present both diagnostic and therapeutic challenges. In this report, we describe a rare case of a rotator cuff intratendinous cyst, not visible on MRI but identified during shoulder arthroscopy. This pathology may help explain persistent symptoms in an otherwise healthy patient.

## Case presentation

A 32-year-old man with right-hand dominance, presented with a two-year history of right shoulder pain. Symptoms occurred while playing darts with no history of trauma. He experienced pain over his right shoulder, especially with forward flexion and overhead activities. Initial physical examination revealed positive belly press and Gerber lift-off tests, with reduced subscapularis muscle power. The strength of the other rotator cuff muscles was good, and provocative tests were negative. There was tenderness over the bicipital groove. His shoulder's active range of motion was otherwise full. Neer sign and Hawkin’s test for shoulder impingement were negative. O’Brien’s test was also negative.

**Figure 1:**
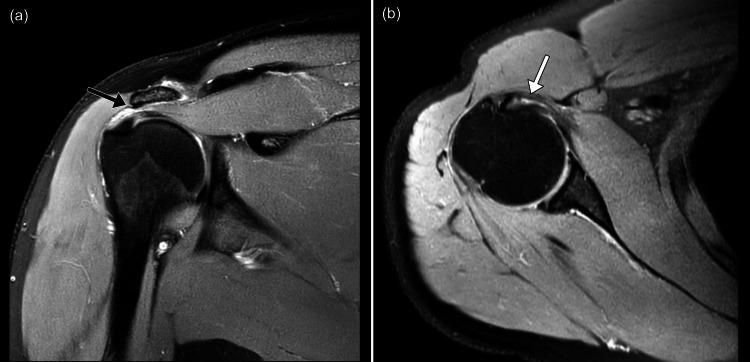
MRI of the right shoulder, proton density weighted images, (a) coronal view and (b) axial view. Black arrow: acromial spur impinging on supraspinatus tendon; white arrow: intrasubstance tendinosis of the subscapularis tendon.

An MRI of his right shoulder was performed. There was an acromial spur impinging onto the supraspinatus tendon, with a partial tear of the tendon at its musculotendinous junction on the bursal side. There was also a low-grade intrasubstance tear of the subscapularis tendon with underlying tendinosis (Figure 1). Other rotator cuff muscles and labrum were normal.

Despite physiotherapy, there was no significant improvement in his symptoms. A repeat clinical examination revealed a full shoulder range of motion with negative provocative tests. However, he had persistent shoulder pain upon flexion and abduction. Although he was still able to perform his daily activities, he was unable to return to competitive dart playing. There was no history of intra-articular shoulder injection or other joint pain.

**Figure 2:**
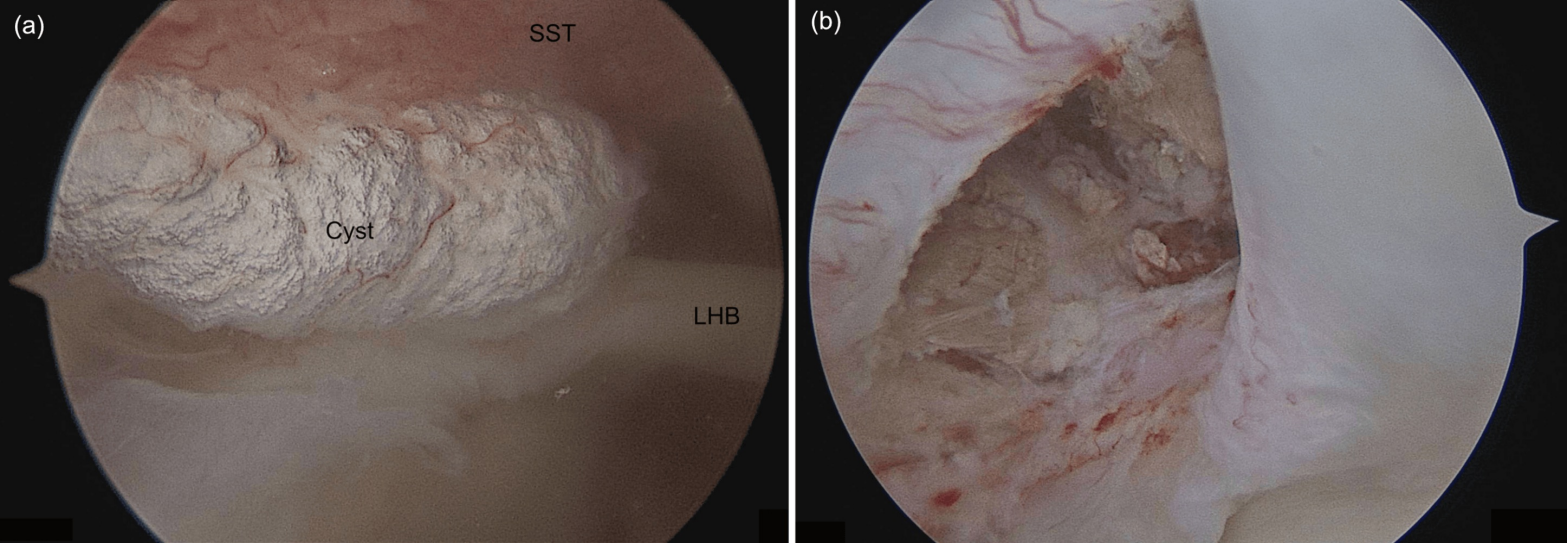
Intraoperative arthroscopic view of the right shoulder showing a supraspinatus cyst visualized on the articular side, (a) pre-debridement and (b) post-debridement. SST: supraspinatus tendon; LHB: long head of biceps tendon.

He was then taken to surgery and a shoulder arthroscopy was performed. Intraoperative findings noted an inflamed long head of biceps tendon, a superior labral anterior-posterior (SLAP) tear grade I, a partial articular supraspinatus tendon avulsion (PASTA) lesion Ellman grade I, and an intratendinous cystic lesion found within the supraspinatus tendon (Figure 2). The cyst measured approximately 10 x 3 x 3 mm. A Bigliani type I acromion with inferior bony spur was present, along with subacromial bursitis. Acromioplasty and biceps tenodesis were performed. The supraspinatus cyst was debrided with arthroscopic shaver and histological examination showed fibro-collagenous tissue lined by synovial cells.

Postoperatively, he was kept in an arm sling for six weeks and initiated on pendulum exercise. At two weeks, passive range of motion exercises were begun for his shoulder and elbow. After six weeks, gradual stretching and strengthening exercises were started. Six months after surgery, he achieved a full shoulder range of motion with a resolution of symptoms.

## Discussion

Fluid collections around the shoulder are often found on MRI or shoulder arthroscopy during investigation for subacromial impingement or rotator cuff pathology. These fluid collections can be classified as bursae or cysts, depending on the histological features of their walls, their origins, or anatomical location.

Bursae, such as the subacromial-subdeltoid bursa and subcoracoid bursa, are typically located between two different tissue types in contact with each other. In normal conditions, they contain a small amount of serous fluid and act to reduce friction between the contact surfaces, such as between tendons or muscles with bone. However, conditions such as inflammation or rotator cuff pathology can lead to an increase in bursal fluid, often visible on MRI.

Cystic fluid collections can be broadly divided into ganglion cysts or synovial cysts. Ganglion cysts are lined by connective tissue and rarely communicate with joints, while synovial cysts are lined by synovial cells and may communicate with the glenohumeral joint [2].

Paralabral cysts, for example, typically develop following a labral tear, with subsequent formation of a one-way valve which allows joint fluid to escape into the adjacent peri-capsular soft tissue. They are usually small and localized, confined to the anterior and posterior glenoid rim by adjacent muscles. While small paralabral cysts may resolve spontaneously, they can also enlarge, typically into the posterosuperior or anteroinferior aspect of the glenoid labrum [6]. If large enough, these cysts cause compression of the suprascapular nerve or axillary nerve, causing significant symptoms [7].

Less common cysts include acromioclavicular joint cysts and rotator cuff cysts. An acromioclavicular joint cyst occurs in the setting of a chronic full-thickness rotator cuff tear, where the inferior capsule of the acromioclavicular joint is disrupted, allowing communication between the glenohumeral joint and the acromioclavicular joint. The MRI appearance of this cyst is referred to as the geyser sign [8].

Intramuscular cysts are usually incidental findings found in association with rotator cuff tears. It is thought to arise as a result of delaminating tears within rotator cuff tendons, allowing glenohumeral joint fluid to enter into the muscle sheaths. These cysts may form within the muscle of the affected rotator cuff tendon, or even from an adjacent torn rotator cuff tendon. Hence, the presence of intramuscular cysts is thought to be an important predictor of rotator cuff tears, especially partial thickness tears [9].

In contrast, intratendinous cysts are extremely rare. To the best of our knowledge, only a few cases have been reported, including two involving intratendinous ganglion cysts of the long head of the biceps tendon and one involving the supraspinatus tendon [10,11]. Siebachmeyer et al. described a persistent articular-sided intratendinous supraspinatus cyst as a cause of shoulder impingement, following repair of a labral tear. Tthe patient’s symptoms resolved only after arthroscopic debridement of the cyst [11]. In our case, the intratendinous cyst was not visible on preoperative MRI but was only discovered intra-operatively during shoulder arthroscopy. This highlights the diagnostic challenge posed by such cysts, as they may not be readily detectable in imaging studies.

The exact etiology of the intratendinous cyst remains unclear. It is postulated to result from tenosynovitis or repetitive mechanical trauma, leading to cystic degeneration of the tendon [12]. In general, treatment depends on symptoms and the location of the cyst. Asymptomatic cysts may resolve spontaneously with observation. However, symptomatic lesions, particularly those causing pain and nerve compression may require intervention such as aspiration or surgical excision. In our case, the cyst was debrided intra-operatively to address the patient’s long-standing pain upon shoulder motion.

## Conclusions

Intratendinous supraspinatus cysts are extremely rare. They can be an overlooked cause of persistent shoulder pain in patients with rotator cuff pathology or subacromial impingement. They present a diagnostic challenge as clinical symptoms may be non-specific and imaging findings can be inconclusive. These cysts should be looked for in an MRI or diagnostic arthroscopy performed for patients with unresolved symptoms despite conservative treatment. This case highlights the importance of considering intratendinous cysts as a potential source of persistent pain. In these cases, surgical cyst debridement can provide significant symptom relief and restore function.
